# A case of alveolar echinococcosis in the liver that ruptured into the pericardium treated by a combination of hepatectomy and albendazole

**DOI:** 10.1186/s40792-022-01417-6

**Published:** 2022-04-08

**Authors:** Yoichi Yamamoto, Yuzuru Sakamoto, Toshiya Kamiyama, Akihisa Nagatsu, Yoh Asahi, Tatsuya Orimo, Tatsuhiko Kakisaka, Hirofumi Kamachi, Takuya Otsuka, Tomoko Mitsuhashi, Akinobu Taketomi

**Affiliations:** 1 Department of Gastroenterological Surgery I, Hokkaido University Hospital, North 14, West 5, Kita-ku, Sapporo, 060-8648 Japan; 2 Department of Surgical Pathology, Hokkaido University Hospital, North 14, West 5, Kita-ku, Sapporo, 060-8648 Japan

**Keywords:** Alveolar echinococcus, Cardiac, Liver, Surgery, Albendazole

## Abstract

**Background:**

Alveolar echinococcosis (AE) is a rare parasitic disease caused by the larva of *Echinococcus multilocularis*. It nearly always occurs in the liver, and cardiac involvement is extremely rare. Liver resection is the most effective intervention for AE because the only potentially curative treatment is removal of the lesion. Even when complete resection is not performed, long-term survival can be expected after surgical removal of most of the lesion with lifelong administration of albendazole (ABZ).

**Case presentation:**

A 64-year-old man who lived in Hokkaido was referred to our hospital due to abnormalities in biliary enzymes. According to the findings from enhanced computed tomography and magnetic resource imaging of the abdomen, transthoracic echocardiography and serologic tests, he was diagnosed with hepatic AE with rupture into the pericardium. He underwent extended left hemi-hepatectomy with reconstruction of the inferior vena cava and opening of the pericardium with drainage as reduction surgery. Pathological examination revealed echinococcal infection in the pericardium as well as the liver. He started chemotherapy with 400 mg ABZ per Day 67 days after surgery. Although the surgical margin was positive in the pathological findings, he was alive 19 months later with no regrowth of the echinococcal lesion.

**Conclusion:**

AE with cardiac involvement is extremely rare. Even if the complete removal of cardiac-involved AE is not possible, surgical debulking with lifelong ABZ treatment can successfully manage the disease.

## Background

Alveolar echinococcosis (AE) is a rare parasitic disease caused by *Echinococcus multilocularis*. This parasite introduces various patterns of hepatic lesions, and it is somewhat different from echinococcosis caused by *Echinococcus granulosus*, a hydatid species that is common worldwide [1]. Data from patients with single-organ involvement indicate that metacestodes initially establish themselves almost exclusively in the liver (approximately 99% of cases) and are rarely found in extrahepatic sites [2]. Later in the infection, metacestodes may spread from the liver to adjacent locations (abdomen, retroperitoneum, etc.) or to distant organs (lungs, brain, bones, etc.) [3]. Cardiac involvement of AE is very rare. It was reported that cardiac involvement AE may result from the direct extension of primary lesions located at the liver dome or from cysts entering the thorax via transdiaphragmatic lymphatic and vascular pathways that then rupture into the pleural cavity or mediastinum [4]. Once cardiac involvement occurs, the patient suffers from risks of arrhythmia, myocardial infarction, and cardiac tamponade, which are difficult to manage.

We present the case of a 64-year-old man from Hokkaido, Japan’s northernmost island, where AE is endemic, who was diagnosed with hepatic AE infiltrating the pericardium and was successfully treated with liver resection and adjuvant albendazole (ABZ). To our knowledge, this is the first case report of hepatic AE with cardiac involvement treated with liver resection and adjuvant ABZ.

## Case presentation

A 64-year-old man was referred to our hospital with abnormalities in biliary enzymes. He lived in Hokkaido, Japan’s northernmost island, and had not been treated for chronic diseases, nor was he taking any medications permanently. He had a history of drinking well water. Although serum enzyme-linked immunosorbent assay (ELISA) with a hydatid cyst antibody was falsely positive for echinococcal infection, the Western blot examination indicated echinococcal infection (Table 1). Enhanced computed tomography (CT) scan showed a 19-cm cystic lesion with calcification in the liver. He was diagnosed with AE of the liver based on serological and radiological findings, and elective surgery was scheduled.

**Table 1:** Laboratory data of the patient

Peripheral blood counts		Biochemistry		Serology	
WBC	11,100/µL	TP	7.8 g/dL	CRP	4.48 mg/dL
Neut	54.6%	Alb	3.1 g/dL	HBsAg	(−)
RBC	492 × 10^4^/µL	T-bil	0.7 mg/dL	HCVAb	(−)
Hb	14.4 g/dL	D-bil	0.1 mg/dL	Tumour markers	
Hct	42.4%	AST	24 U/I	AFP	2.9 ng/dL
Plt	36.1 × 10^4^/µL	ALT	14 U/I	CEA	6.3 ng/mL
Coagulation		ALP	987 U/I	CA19-9	69.4 U/mL
PT	83.2%	γ-GTP	264 U/I	Others	
APTT	31 s	BUN	12 mg/dL	ELISA	(±)
Fibrinogen	448 mg/dL	Cr	0.5 mg/dL	WB	(+)
d-dimer	0.85 µg/mL				

WBC, white blood cells; Neut, neutrophils; RBC, red blood cells; Hb, haemoglobin; Hct, haematocrit; Plt, platelet count; PT, prothrombin time; APTT, activated partial thromboplastin time; TP, total protein; Alb, serum albumin; T-bil, total bilirubin; D-bil, direct bilirubin; AST, aspartate aminotransferase; ALT, alanine aminotransferase; ALP, alkaline phosphatase; γ-GTP, gamma-glutamyl transpeptidase; BUN, blood urea nitrogen; Cr, creatinine; CRP, C-reactive protein; HBsAg, hepatitis B virus surface antigen; HCV Ab, hepatitis C virus antibody; AFP, alpha-fetoprotein; CEA, carcinoembryonic antigen; CA19-9, carbohydrate antigen 19-9; ELISA, enzyme-linked immunosorbent assay; WB, Western blotting

**Fig. 1:**
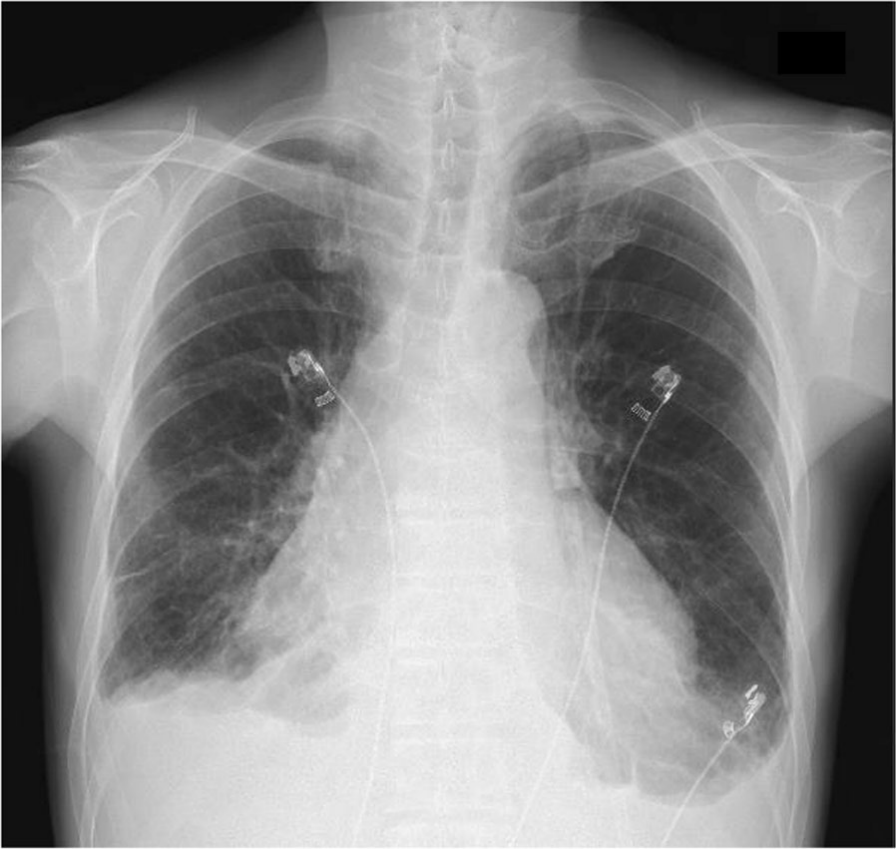
Chest X-ray showed a high cardiothoracic ratio of 63%

**Fig. 2:**
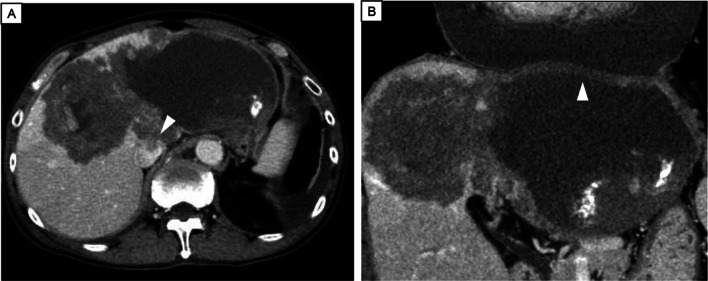
**A** CT showed a hepatic lesion with calcification. White arrow head: the echinococcal invasion of the IVC. **B** Hepatic lesion and pericardial fluid. White arrow head: echinococcal invasion of the pericardium

**Fig. 3:**
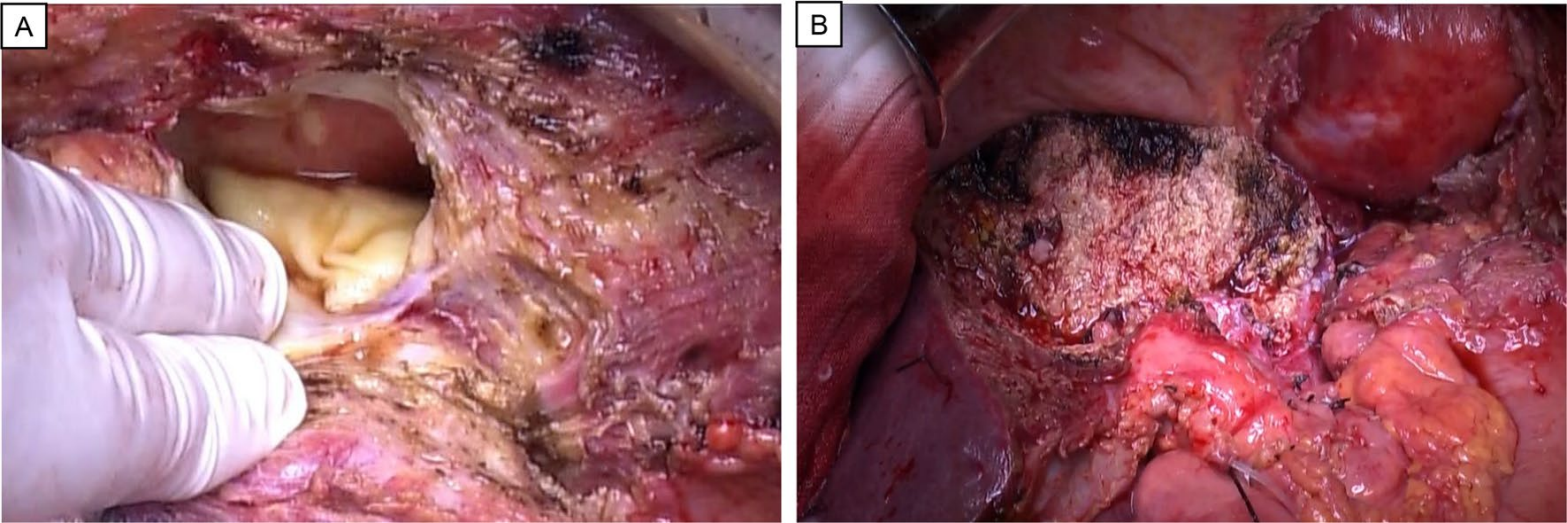
**A** The pericardium was opened, and a white cystic structure was observed. **B** Expanded left-sided hemi-hepatectomy, IVC reconstruction, and pericardial drainage

**Fig. 4:**
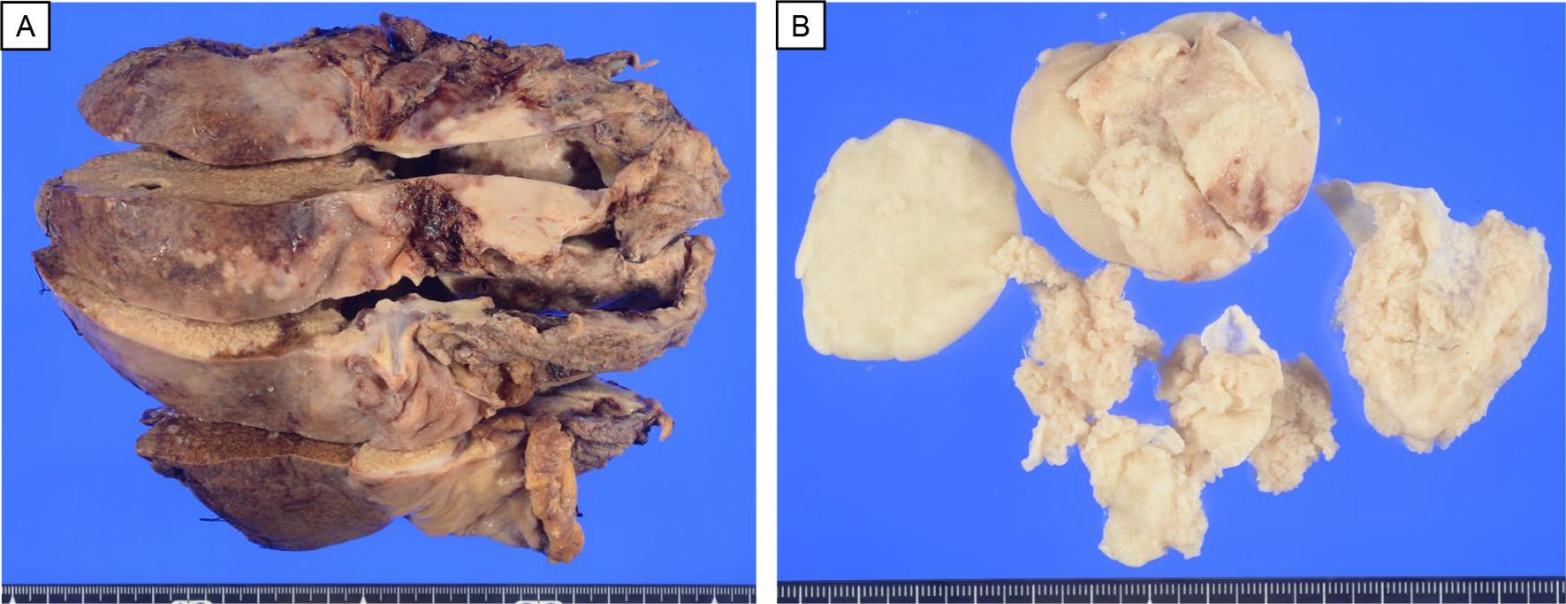
**A** Removal of the left side of the liver. **B.** The white cystic structure removed from the pericardium

**Fig. 5:**
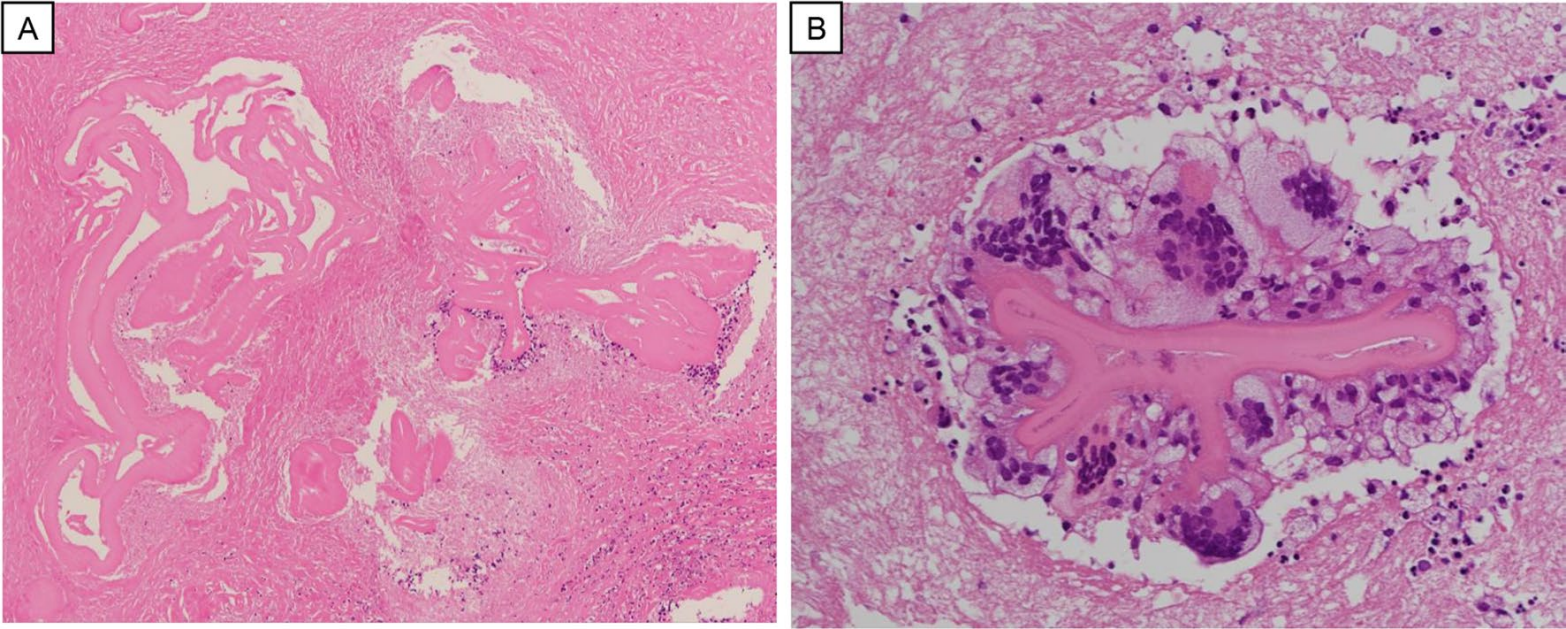
**A** Pathological findings of the liver (×100). The specimens stained with haematoxylin–eosin (H&E) showed alveolar echinococcosis vesicles with laminar membranes. **B** Pathological findings of the cystic structure in the pericardium (×200). The specimens stained by H&E showed alveolar echinococcosis vesicles with laminar membranes

He was admitted to our hospital with complaints of breathing difficulty several days before surgery. He had a body temperature of 37.5 °C and oxygen saturation of 94% on 3 L/min oxygen inhalation. There were no other abnormal findings on physical examination. Chest X-ray showed a high cardiothoracic ratio of 63% (Fig. 1), but electrocardiography revealed a normal sinus rhythm. The CT scan showed cystic lesions not only in the liver, but also in the pericardium, suggesting the AE of the liver had ruptured into the pericardium (Fig. 2). The patient underwent transthoracic echocardiography, which demonstrated pericardial effusion and floating highly echoic material, also suggesting the rupture of the AE into the pericardium. Although we consulted a cardiologist, pericardial drainage was not performed because the patient’s vital signs were stable and there were no significant symptoms suggesting cardiac tamponade. Nine days after admission, the surgery was performed. Because rupture of the AE into the pericardium was expected, cardiac surgeons stood by. Laparotomy with a Mercedes-Benz incision revealed that the cystic lesion in the liver of the left lobe had invaded the diaphragm, pericardium, and inferior vena cava (IVC). First, pericardial drainage was done by cutting down the pericardium (Fig. 3A). After that, extended left hemi-hepatectomy, partial hepatectomy of segment 6, reconstruction of the IVC, and reconstruction of the pericardium were done (Fig. 3B). Drains for the pericardium and Winslow’s foramen were inserted. A cystic duct tube was also inserted in case of postoperative bile leakage. The operation time was 7 h and 23 min, the amount of bleeding was 1710 mL, and 560 g of red blood cells and 720 g of fresh frozen plasma were transfused. On pathological findings, the specimen (Fig. 4) with haematoxylin–eosin staining (Fig. 5) showed alveolar echinococcosis vesicles with laminar membranes in a pericardial cavity excision. Upon liver resection, the diagnosis of AE of the liver that had ruptured into the pericardium was confirmed. The surgical margin of the hepatic left lobe was positive, suggesting a remaining echinococcal lesion in the liver. The drains for the pericardium and Winslow’s foramen were removed on postoperative days (PODs) 4 and 5, respectively. The complications of bile leakage and abscess on the hepatic resection surface occurred, which required percutaneous drainage treatment on POD 11. The drain for the abscess was removed on POD 36. The cystic duct tube remained in place for 20 months after surgery because of bile leakage. Although the complications extended his hospital stay, he was discharged 7 weeks after the surgery. There were no cardiac complications after surgery. Because the surgical margin was positive, he was put on medical treatment with 400 mg ABZ per day, and no regrowth of the remaining lesion was confirmed at 19 months. As long as the remaining lesion does not grow, he will take ABZ for life. If the lesion does regrow, we will consider re-excision of the lesion if his liver function and operability allow it.

## Discussion

Echinococcosis is the general name given to zoonotic infections caused by tapeworms (cestodes) of the genus Echinococcus. The life cycle of this parasite occurs in two different mammalian organisms (definitive host and natural intermediate host). Humans are accidental or aberrant intermediate hosts and are not a part of the natural life cycle of the parasite [3]. Of the three forms of echinococcosis occurring in humans, cystic echinococcosis (CE) and AE are of special importance due to their wide geographic distribution and their medical and economic impact. Polycystic echinococcosis is less frequent and is restricted to Central and South America [3]. Alveolar echinococcosis occurs only in the Northern Hemisphere. Most human cases of *E. multilocularis* infection have been reported in endemic areas of western and central Europe, including Turkey, Russia, Iran, Iraq, western and central China, and northern Japan (Hokkaido island) [5].

AE is a serious disease with a greater than 90% mortality rate in untreated patients [6]. The symptomatology of AE depends on the affected organ, cyst size and location of the cyst expanding interaction with adjacent organs. AE most often affects the liver (98% of all cases), and the infection is usually clinically silent for many years [7]. The clinical symptoms usually develop after a long incubation period (5–15 years) [3]. Without adequate treatment, AE grows slowly and progressively in the liver, which is why treatment with curative intent should be attempted whenever possible [8]. Large cysts resemble invasively growing tumours and may cause abdominal pain, jaundice, and hepatic vein thrombosis with portal hypertension [7]. For hepatic AE, hepatectomy offers the only curative treatment if the lesions are completely resectable at their location of origin and liver function would be well preserved by this procedure [9]. In many cases, unfortunately, complete resection is not feasible at the time of diagnosis. In the case of hepatectomy for a large AE lesion, it is important to drain the cyst and have a good surgical view. Determining the placement of the IVC, hepatic vein, portal vein, hepatic artery, and bile duct before surgery and not hesitating to convert to reduction surgery are also important. Even in cases in which complete resection is not feasible, reduction surgery followed by ABZ therapy can lead to long-term survival. According to the report by Kawamura et al., a 92.8% rate of 15-year overall survival was achieved in patients with reduction surgery (more than 90% reduction of the lesion) followed by lifelong ABZ therapy [10]. Because our patient had a positive surgical margin, he will take ABZ for life.

Hepatic AE with cardiac involvement is extremely rare. It can occur by two pathways: direct invasion and haemodynamic metastasis. In our case, direct invasion from the hepatic lesion through the diaphragm was suggested by the radiological findings. To the best of our knowledge, there are only four reported cases of hepatic AE with cardiac involvement (Table 2) [11–14]. The reported cases were treated with surgery alone or ABZ alone.

**Table 2:** Reported hepatic AE with cardiac involvement

Year	Author	Age/sex	Cardiac lesion	Other lesion	Surgery	Chemotherapy	Outcome
1980	Khuroo MS	29/male	Rt. atrium	IVC	Done	No	Death (6 POD)
1986	Etievent JP	64/female	Rt. atrium	IVC, lung	Done	No	Alive at 42 months
2012	Kantarchi	39/male	Pericardium Rt. ventricle	Not identified	Not identified	Not identified	Not reported
2020	Neettu	7/not identified	Rt. atrium	IVC	No	ABZ	Death (2 months)
2021	(current)	64/male	Pericardium	IVC	Done	ABZ	Alive at 20 months

Rt, right; IVC, inferior vena cava; ABZ, albendazole

The first case of cardiac alveolar echinococcosis was recently published [15]. The patient was a 31-year-old woman who had cardiac AE in the right atrium with IVC invasion and cerebral lesion. Open-heart surgery was performed, and the patient was alive after more than 1 year. This result suggests the efficacy of surgical treatment for cardiac lesions.

This is the first report of hepatic AE with cardiac involvement treated by the combination of surgery and ABZ. Even in cases like this, a surgical procedure with postoperative ABZ can be a useful treatment.

## Conclusion

This is the first report of (extremely rare) hepatic AE with cardiac involvement that was treated with surgery and ABZ. Even in the case of cardiac involvement, surgery followed by ABZ can be a promising treatment.

## Data Availability

Not applicable.

## Abbreviations

ABZ: Albendazole
AE: Alveolar echinococcosis
CE: Cystic echinococcosis
CT: Computed tomography
ELISA: Enzyme-linked immunosorbent assay
IVC: Inferior vena cava
MRI: Magnetic resonance imaging
POD: Postoperative day
